# End-to-End Automatic Pronunciation Error Detection Based on Improved Hybrid CTC/Attention Architecture

**DOI:** 10.3390/s20071809

**Published:** 2020-03-25

**Authors:** Long Zhang, Ziping Zhao, Chunmei Ma, Linlin Shan, Huazhi Sun, Lifen Jiang, Shiwen Deng, Chang Gao

**Affiliations:** 1 College of Computer and Information Engineering, Tianjin Normal University, Tianjin 300387, China; zhanglong@tjnu.edu.cn (L.Z.); zhaoziping@tjnu.edu.cn (Z.Z.); machunmei@tjnu.edu.cn (C.M.);; 2 College of Fine Arts and Design, Tianjin Normal University, Tianjin 300387, China; 3 School of Mathematical Sciences, Harbin Normal University, Harbin 150080, China; 4 School of Information Science and Engineering, Yanshan University, Qinhuangdao 066004, China

**Keywords:** automatic pronunciation error detection, ASR, CTC, attention-based, seq2seq model, end-to-end, CAPT

## Abstract

Advanced automatic pronunciation error detection (APED) algorithms are usually based on state-of-the-art automatic speech recognition (ASR) techniques. With the development of deep learning technology, end-to-end ASR technology has gradually matured and achieved positive practical results, which provides us with a new opportunity to update the APED algorithm. We first constructed an end-to-end ASR system based on the hybrid connectionist temporal classification and attention (CTC/attention) architecture. An adaptive parameter was used to enhance the complementarity of the connectionist temporal classification (CTC) model and the attention-based seq2seq model, further improving the performance of the ASR system. After this, the improved ASR system was used in the APED task of Mandarin, and good results were obtained. This new APED method makes force alignment and segmentation unnecessary, and it does not require multiple complex models, such as an acoustic model or a language model. It is convenient and straightforward, and will be a suitable general solution for L1-independent computer-assisted pronunciation training (CAPT). Furthermore, we find that in regards to accuracy metrics, our proposed system based on the improved hybrid CTC/attention architecture is close to the state-of-the-art ASR system based on the deep neural network–deep neural network (DNN–DNN) architecture, and has a stronger effect on the F-measure metrics, which are especially suitable for the requirements of the APED task.

## 1. Introduction

With the continuous development of economic globalization and social integration, more and more people are eager to learn a second language. The computer-assisted language learning (CALL) system, which can provide flexible self-paced learning anytime and anywhere, and cheaper and more immersive learning with real-time feedback and personalized guidance, is becoming more and more popular. CALL systems that focus on speech and pronunciation are usually called computer-assisted pronunciation training (CAPT) systems. CAPT systems can efficiently process and analyze the speech uttered by language learners and then provide the quantitative or qualitative assessment of pronunciation quality or ability to them as feedback. This process is also known as the automatic pronunciation (quality/proficiency) assessment (evaluation/scoring). More to the point, CAPT systems should be able to accurately detect pronunciation errors in the utterances produced by language learners, diagnose the types and locations of pronunciation errors, and then provide corrective feedback and operational guidance for improvement. Thus, it can be deduced that automatic pronunciation error detection (APED) is the core of CAPT systems. APED is also referred to as mispronunciation detection and diagnose (MD or MDD) in some literature [1,2,3].

**Figure 1:**
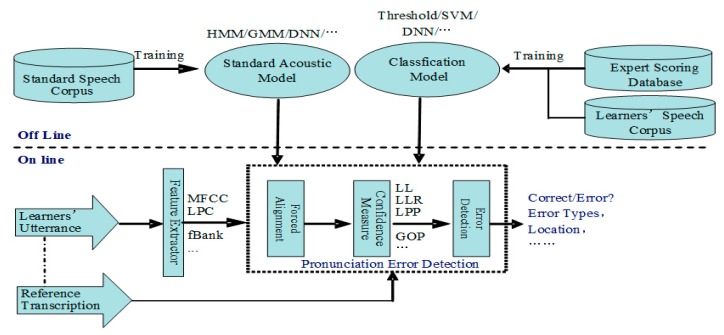
Framework of a typical automatic pronunciation error detection (APED) system.

Presently, the advanced APED is mainly based on the state-of-the-art automatic speech recognition (ASR) technique and has made steady progress with the development of the ASR. As shown in Figure 1, the framework of a typical APED system is as follows: first, force alignment is applied, in which the sequence of acoustic frames of language learners’ utterances is fixed by the sequence of phone models derived from the reference (prompt) transcription of the utterance, with a standard speech recognizer trained in advance through the standard speech corpus; then the likelihood or probabilities of the force alignment segments are calculated as confidence measures, which indicate how similar the pronunciation is to the canonical pronunciation; finally, a classifier is also constructed through language learners’ speech corpora and the corresponding expert scoring database, which uses confidence measures and other speech features (e.g., phone duration, also obtained by force alignment) to judge whether the pronunciation is correct or not [4]. The effective confidence measures usually include logarithm likelihood (LL), logarithm likelihood ratio (LLR), logarithm posterior probability (LPP), normalization logarithm likelihood ratio (NLLR), goodness of pronunciation (GOP), and more [5].

The construction of an APED system is very complex. The first step is to construct a sophisticated ASR system, and then a classifier or model for the identification of pronunciation errors, which is trained by using the speech corpora containing pronunciation errors and the expert scoring database, annotating the errors.

An ASR system can be implemented by several different technologies, including the earliest dynamic time warping (DTW) and vector quantization (VQ), the classical gaussian mixture model–hidden Markov model (GMM–HMM), and the now-most popular deep neural network-hidden Markov model (DNN–HMM), DNN–DNN, and various neural network (NN) models within a deep learning framework [6,7,8]. Classifiers for an APED task can also be built by some models, for example, the early decision tree (DT), the classical support vector machine (SVM) and ensemble learning (EM), and the most popular NN. When the classifier cannot be trained without the expert annotated data, APED can also be achieved by setting thresholds directly in accordance to confidence measures [4]. With the continuous development of ASR technology, the accuracy of ASR is so high that some studies directly detect pronunciation errors based on results of the ASR and achieve good performance in the APED task [9].

Observing the framework of the APED system, we can see that there are many factors affecting the performance. As such, the related works focus on the following aspects: (1) improving the calculation method of the confidence measure; (2) discriminative training to improve the accuracy of the acoustic model; (3) using a more refined DNN-based acoustic model; (4) selecting more distinctive features; (5) building better classifiers. We will introduce them in detail in Section 2.

However, there are some problems in the above work. First, a basic GMM–HMM acoustic model must be trained, and then it is used to make force alignment and segmentation of speech based on the reference transcription. If segmentations are wrong at the beginning, confidence measures, extracted features, trained classifiers, and so on become inaccurate, or even completely wrong. Moreover, due to the diversity and complexity of many aspects, such as application environment, recording devices, speakers’ voices (especially the non-native pronunciation of second language learners), it is usually challenging to achieve accurate segmentation. Force alignment segmentation, which not only depends heavily on the accuracy of speech recognition model but also above factors, has become an inevitable bottleneck in the APED system.

With the development of deep learning technology, end-to-end ASR technology has gradually matured and achieved positive practical results, which provides us with a new opportunity to update the APED algorithm. Traditional ASR consists of many modules, including the acoustic model, lexicon model, language model, and more. It also requires linguistic resources, such as a handcrafted pronunciation dictionary to map word to phone sequences, tokenization for some languages without the explicit word boundary, and phonetic context dependence trees. It is especially challenging to build an ASR system for a new language. Moreover, each module in an ASR system needs to be optimized independently, and their optimization objective functions are inconsistent with the overall goal of the task. In addition, because there are many modules, the error in the previous module has a significant impact on the subsequent module. End-to-end ASR can simplify many modules in traditional ASR into a single-network architecture within a deep learning framework and solve the above problems well. It is a unified model which is simple and direct, and the whole training process does not require forced alignment and segmentation.

At present, there are two major architectures for end-to-end ASR, one is the connectionist temporal classification (CTC) model [10,11], and the other is the attention-based Seq2seq model [12,13]. Among them, the CTC model uses the Markov hypothesis to solve sequential problems effectively through dynamic programming, and the attention-based model adopts attention mechanism to perform alignment between acoustic frames and recognizable symbols. The most significant advantage of end-to-end ASR is that it abandons a series of assumptions of traditional HMM-based ASR, and no longer requires forced alignment and segmentation, and has a maximum likelihood of training speech segments. It does not even require language models, and simplifies the ASR system by using a single network architecture to represent complex modules in traditional ASR. It greatly reduces the difficulty of building an ASR system.

These two kinds of end-to-end ASR methods have their own advantages and disadvantages. The CTC method is more geared to time series modeling and the attention-based method does not need to satisfy the independence assumption. However, the attention-based method is too flexible to guarantee the order of output sequences, which is defective for the ASR task. To utilize the advantages of both methods, a hybrid CTC/attention architecture for end-to-end ASR was proposed [14,15]. In the training process, the multi-objective learning (MOL) framework was used to improve robustness and achieve fast convergence. In the decoding process, two objective functions were interpolated linearly by a hyper-parameter and then a joint decoding using an optimized objective function was employed in a one-pass beam search algorithm to further eliminate irregular alignments. However, this hyper-parameter needed to be set manually before decoding and kept constant throughout the decoding process. In Section 3, an adaptive parameter is proposed instead of the hyper-parameter. The value of the adaptive parameter can be adjusted continuously according to the current values of two loss functions in the whole decoding process, and therefore alignment processing will be better.

In this paper, an end-to-end APED system based on improved hybrid CTC/attention architecture was constructed, and then the performance of the system was further evaluated. This APED system based on hybrid CTC/attention architecture is very innovative and promising, as it does not require force alignment segmentation and language models.

The contributions of this paper are as follows:

1. From the perspective of the technical development of ASR, we carefully review the classical models and technical methods of APED technology over the past 20 years, and then observe the monumental APED systems at different periods as baseline systems to analyze their advantages and disadvantages and to evaluate their performance.
2. To solve the problem of the empirical parameter of the traditional end-to-end ASR based on hybrid CTC/attention architecture needing to be set manually before the training and remaining unchanged throughout the training process, we introduce an adaptive parameter based on the Sigmoid function that does not need to be set in advance and can be adjusted continuously during training. It can make full use of the advantages and disadvantages of the CTC model and the attention-based model, and helps estimate the alignment process better. In the ASR task of Mandarin, the improved system with an adaptive parameter achieved better recognition results, which is superior to all the traditional systems with different manual parameters.
3. We use the improved ASR system in the APED task of Mandarin and obtain a result. To the best of our knowledge, there are no previously published results for the end-to-end APED system. The end-to-end APED based on improved hybrid CTC/attention architecture does not require segmentation by force alignment and multiple complex models. It is convenient and straightforward, and will be a suitable general solution for L1-independent CAPT.

The rest of this paper is organized as follows: The related works for the APED task are introduced in detail from the perspective of ASR technology in Section 2; In Section 3, a new and promising APED system based on improved hybrid CTC/attention architecture is proposed; In Section 4, we present the results obtained from the experiments and discuss them; Finally, the conclusion along with future work based on our research findings is shown in Section 5.

## 2. Literature Review

There are two ways to build an APED system. One is based on ASR technology, and the other is based on acoustic phonetics. The ASR-based approach regards the problem of APED as the problem in the calculation confidence measures where phones (or other basic pronunciation units) can be correctly recognized by a standard ASR system, that is, the confidence measure of signal X decoded into pattern P. Based on this idea, the goal of an APED system is to find effective confidence measures and combined features, which can produce higher scores for standard pronunciation, but lower scores for non-standard pronunciation. If these scores are lower than certain thresholds, they can be detected as pronunciation error, or these scores are fed into the trained classifier to determine whether the pronunciation is correct. The approach based on acoustic phonetics usually regards the problem of APED as the problem of comparison or classification. Therefore, based on the statistical analysis of phonetics, it first extracts all kinds of features at the segment, including acoustic features, perceptual features, and structural features, and then finds discriminative features or combined features from them. Finally, using these features, an advanced classifier or comparator is built for a specific APED task on a specific set of phones.

### 2.1. APED Methods Based on ASR Technology

The APED method based on ASR technology focuses on the following three aspects: constructing and optimizing the calculation of confidence measures, improving the adaptability and evaluation performance of the acoustic model, and refining the acoustic model by deep learning technology.

#### 2.1.1. Confidence Measure and Its Improvement

The basic confidence measure is derived from the probability that a GMM–HMM-based phone acoustic model is able to generate the phonetic segmentation according to intermediate results obtained in the decoding process of an ASR system.

In 1996, Neumeyer L of the Stanford Research Institute (SRI) first proposed the confidence measure in a pronunciation quality assessment, which was based on HMM logarithmic likelihood. However, the experimental results were not satisfactory, and the correlation with the expert scores is worse than the normalized length scores of phonetic segmentation [16]. In 1997, Franco H of SRI proposed a new confidence algorithm, with the logarithmic posterior probability based on HMM. The experimental results in the speaker levels and sentence levels show that the new algorithm was clearly better than other confidence measures [17]. Kim Y extended the above research to the phone level. The correlation between logarithmic posterior probability based on HMM and the expert scores was the best, but there is still a big gap between this correlation and the correlation at speaker level and sentence level, which indicates that the confidence measure is not reliable enough at the phone level alone [18].

Over the same period, Witt S M of the University of Cambridge (CU) conducted a phone-level mispronunciation diagnosis study. Goodness of pronunciation (GOP) was proposed as a confidence measure, and a predefined threshold was used to determine whether the pronunciation was correct [4,19]. The literature [20] outlined a detailed analysis of the performance of GOP algorithm under various application conditions. The experimental results showed that the GOP algorithm is excellent in adaptability and stability, and it has low requirements for speaker and threshold. Nowadays, the GOP algorithm and its improved algorithm are widely used in most APED systems.

Aiming at the shortcomings of classical GOP algorithm in the method of computation, Song presented a lattice-based GOP algorithm utilizing the information from the lattice of ASR, and generally found better results than in the classical GOP except for the APED of short sentences [21]. Zhang expanded the standard pronunciation space to include pronunciation errors through an adaptive unsupervised clustering algorithm, and then refined more detailed acoustic models for APED within the extended pronunciation space (EPS). If the EPS is large enough and models all types of pronunciation errors of each phone, the APED within the EPS not only produces a better result, but also points out the locations and types of pronunciation errors [22].

#### 2.1.2. L1-Dependent Confidence Measure

In the L1-dependent L2 APED task, researchers utilized non-native corpora to construct learners’ typical pronunciation conversion rules (L1/L2 error patterns) to build a dictionary of pronunciation variation or a network of pronunciation variation. After this, each phone, and phones that are easily confused with those phones, are processed uniformly in a decoding process or in a multi-level system. Usually, these L1-dependent methods can improve the accuracy of detection.

Wang analyzed the differences between Cantonese and American English from the perspective of cross-linguistics, summed up the rules of pronunciation errors generated by Cantonese, and built a dictionary of pronunciation variations containing all possible errors. To remove the unreasonable pronunciation in the dictionary, an efficient pruning algorithm was used to modify the dictionary through the confusion network in the training set. Utilizing the dictionary learners’ pronunciation errors could be detected quickly and accurately [23]. Meng established CUCHLOE (Chinese University Chinese Learners of English) corpora. Through the comparative analysis and error analysis of non-native speakers’ accents and standard native speakers’ pronunciation, the typical error patterns of Cantonese speakers in English were obtained. These error patterns were used to expand the recognition network and generate a pronunciation variation network, which could fix the positions of pronunciation errors and give some advice for correct pronunciation [24,25,26].

The above methods of automatically generated pronunciation conversion rules (pronunciation variation dictionary or pronunciation variation network) often lead to the expansion of error coverage and an increase in complexity. Therefore, Kawahara T. proposed a decision tree-based method to directly generate a speech recognition grammar network, which achieved better results in the experiment of foreign students learning Japanese [27]. Stanley directly applied machine translation technology to automatically construct L1 pronunciation error patterns, which significantly improved the precision and recall rate of pronunciation errors and had similar accuracy with the method based on pronunciation conversion rules [28].

The L1-dependent confidence measures can make use of the typical pronunciation errors of language learners from different countries or regions to the greatest extent possible. It is targeted more in the pronunciation quality assessment and helps to improve the performance of the assessment method. However, this method cannot cover all possible errors. The corresponding pronunciation dictionary or pronunciation conversion rules need to be adjusted according to the application scenarios. It relies heavily on prior knowledge and has obvious shortcomings. It is more suitable for the application tasks that only need to detect typical pronunciation errors.

#### 2.1.3. Improved Acoustic Model

In addition to confidence measures, the ways in which the adaptability and discriminability of the acoustic model for APED tasks could be improved has also been widely concerned.

Witt analyzed the similarities and differences in the frequency spectrum, time duration, and pronunciation style between native and non-native speakers. The speaker adaptive technology was introduced to adjust the mean of the model, which reduced the mismatch between the acoustic model and the speakers and improved the speech recognition of non-native speakers [29]. Ohkawa used bilingual speakers’ utterances to adapt L1 and L2 acoustic models and trained multiple bilinguals’ models for the CALL system. Through these methods, the system performance was improved by 5% to 10%, respectively [30]. Song Y et al. used three strategies to get a better standard acoustic model. One was to regulate the changes between speakers through speaker adaptive training, the other was to improve the distinction between confusing phones by minimizing phone error training, and the third was to compensate for the difference of accent between L1/L2 by maximum likelihood linear regression (MLLR). Finally, the correlation of man–machine scoring increased from 0.651 to 0.679 at sentence level and increased from 0.788 to 0.822 at speaker level [31]. To avoid over-adaptation and improve the fault tolerance of MLLR, Luo D., a Japanese scholar, proposed a regularized MLLR transformation method that used a group of teachers’ data to regularize learners’ transformation matrices. This method assumed that the learners’ transformation matrices were the linear combinations of teachers’ matrices, which theoretically guaranteed that the acoustic model still maintained the golden standard after adaptive transformation. The experimental results also showed that the methods could utilize MLLR adaptation better and have good fault tolerance [32]. Zhang J. et al. trained phone models with different pronunciation qualities by using speech sample data of different pronunciation qualities. By applying force alignment using conventional acoustic models, they decoded the boundary information of the phone and obtained the pronunciation quality grade of the phone directly. At the phone level and sentence level, the results are better than the GOP scores [33].

#### 2.1.4. Acoustic Model Based on Deep Neural Network

DNNs can learn the multi-level abstract representations of input data through their multiple processing layers and have recently made remarkable achievements in many pattern recognition tasks, such as image classification, speech recognition, object detection, and drug discovery [34,35]. In the field of ASR, many kinds of DNNs, including feedforward neural networks [36], convolutional neural networks [37,38], and recurrent neural networks [39,40], are mainly used in acoustic models and used partly in lexicon models and language models [12,41]. They have widely improved the performance of advanced ASR systems.

Qian first modeled the phone-state posteriors in HMMs using the deep belief network (DBN) to replace GMMs in APED. The acoustic models based on the DBN–HMM framework that were trained in an unsupervised manner with additional unannotated L2 data displayed significant improvements but were computationally more expensive [42]. Hu refined acoustic models based on DNN with discriminative training and defined three different GOP scores in the framework of DNN–HMM. The experimental results showed the best GOP, in which DNN was 22% higher than the standard GOP with non-DNN in the correlation of man–machine scoring [43]. In the following research, multiple logistic regression classifiers were integrated into a neural network with shared hidden layers to replace a GOP-based classifier and SVM classifier, which achieved better performances in the APED task [2]. Kun proposed an acoustic-graphemic-phonemic model (AGPM) for the mispronunciation detection, whose acoustic model and state transition model are multi-distribution DNNs. To implicitly model error patterns, acoustic features, graphemes, and phonemes are integrated as inputs of the AGPM. It worked similarly to freephone recognition, but achieved excellent results [9].

### 2.2. APED Methods Based on Acoustic Phonetics

ASR-based methods are the mainstream methods used in the existing APED systems. Their advantages are simple calculations, which can use the intermediate results of speech recognition directly, and their calculation methods, which are the same for all phones. Their disadvantage is that their diagnostic information is not precise enough and lacks more instructive feedback. Acoustic–phonetic-based methods usually extract distinctive features (selection of pronunciation features) for the specified target to be evaluated, and then use DTW algorithms to calculate similarity after force alignment (comparison-based method); or select classifiers to distinguish the pronunciation levels (classification-based method).

#### 2.2.1. Selection of Pronunciation Features

The APED methods based on acoustic phonetics usually aim at specific research tasks, and combine the existing research experience of acoustic phonetics to select a variety of distinctive pronunciation features. Therefore, the selected pronunciation features are often diverse, including time domain features, time–frequency features, auditory model features, short-term spectrum features [44], trap structure [45], speech structure feature [46,47], formant [48], and pronunciation articulatory [49,50]. However, it is not yet clear which features can truly represent the speaker’s pronunciation quality.

#### 2.2.2. Comparison-Based Methods

The earliest method of APED is based on comparison. This method generally uses DTW algorithms to align the speech to be evaluated with the standard speech, and then extracts the corresponding evaluation features, calculates the distance between these features, and finally maps them to the pronunciation quality score according to the distance.

Lee A. proposed a comparative method to detect pronunciation errors at the word level of non-native speech. Through DTW of non-native speech and native speech, word-level and phone-level features that can effectively describe mismatched degree information on matching paths and distance matrices were extracted [51]. Subsequently, the author used the posterior probability of the deep neural network as an input feature, and the performance of the system was improved by at least 10.4%. When only 30% of the labeled data was used, the performance of the system remained stable [52].

#### 2.2.3. Classification-Based Methods

The APED task can essentially be regarded as a classification problem, using a set of scoring features as an input, optimizing some criteria or objective function, and finally classifying them into different pronunciation levels. Therefore, classification-based methods have become the most important methods in the APED task, and various types of classifiers have been widely used, such as DT, SVM, AdaBoost, and NN.

Truong K. carried out the APED task for three phones, /A/, /Y/, and /x/, that are frequently mispronounced by L2-learners of Dutch. By comparing the different acoustic–phonetic features of correct and incorrect pronunciation, some distinguishing features, such as time duration, rate of rise (ROR) maximum, and energy amplitude, were chosen to train and test classifiers. Linear discriminant analysis and decision trees were used to train the classification model respectively, and positive results were obtained in both native and non-native speech [53,54]. Patil V. selected appropriate acoustic–phonetic features, including frication duration, difference between the first and second harmonic, spectral tilt, signal-to-noise ratio, B1-band energy, and more in the APED task on aspirated consonants of Hindi, and showed that acoustic–phonetic features outperform traditional cepstral features [55].

Acoustic–phonetic-based methods are usually aimed at specific APED tasks for some commonly confused phones on small-scale speech corpora, utilizing the abundant knowledge of linguistic phonetics. To find the most discriminative features, and to combine these features to train an efficient classifier for the APED, is the key.

In recent years, the acoustic–phonetic-based methods have been deeply integrated with the ASR-based methods and they have been shown to complement each other. With the help of state-of-the-art ASR technology, the accurate segmentation of multi-level segments on the large-scale corpus and the robust confidence measures are achieved. Discriminative features are constructed by using acoustic–phonetic knowledge and refined acoustic models. These multi-type complementary features feed in a well-structured classifier, thus improving the accuracy of APED in a well-rounded way.

## 3. Proposed Methodology

**Figure 2:**
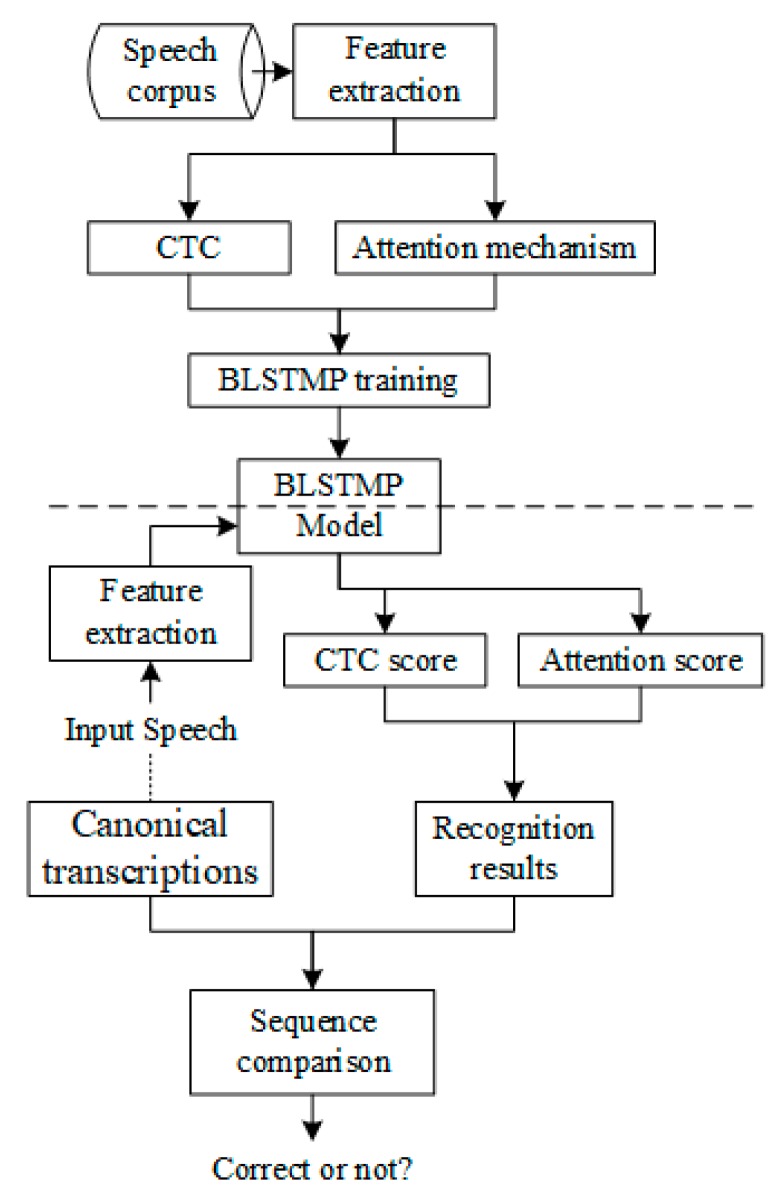
Block diagram of the end-to-end APED system based on hybrid connectionist temporal classification (CTC)/attention architecture.

In this section, we propose a new end-to-end ASR system based on improved hybrid CTC/attention architecture to detect pronunciation errors. The main process of this method is five steps: (1) Data preparation. There is no need to prepare a pronunciation dictionary and a language model, and trained GMM–HMMs and force alignment are not necessary in the stage; (2) Acoustic feature extraction. To extract Mel scale filter bank coefficients and fundamental frequency features from speech waveforms; (3) Encoder and decoder network training using hybrid CTC/attention end-to-end architecture. To reduce the error rate and accelerate the training, bidirectional long short term memory projection (BLSTMP) is selected [56,57]. The encoder network is trained by CTC criterion and the attention mechanism, and the probability of CTC is considered to find more consistent inputs. The CTC probability enforces monotonic alignment in the decoding process and does not allow large jumps or the cycle of the same frame. At the same time, CTC and attention-based probability scores are calculated to obtain robust decoding results; (4) Speech recognition. Recognition results can be obtained by using the end-to-end network models from step 3; (5) Sequence comparison. To compare speech recognition results with canonical transcriptions, the Needleman–Wunsch algorithm [58] can be used to calculate the insertion error, deletion error, and substitution error of the two sequences, and it then can produce the detection of pronunciation errors. The whole process is shown in Figure 2.

Next, we introduce the CTC model, the attention-based model, and the hybrid CTC/attention model in detail.

### 3.1. CTC Model

ASR can be considered the sequence mapping an acoustic observation vector sequence of length T, $o=\left\{o_{t}\in R^{D}|t=1,\cdots ,T\right\}$, to the corresponding word sequence of length *N*, $W=\left\{w_{n}\in V|n=1,\cdots ,N\right\}$. Where $o_{t}$ is the observation vector of the frame t, $w_{n}$ is the $n^{th}$ word of W in the vocabulary, V. The aim of ASR is to evaluate all possible word sequences, $V^{*}$, to find the most likely word sequence, $\hat{W}$.

(1) $$\hat{W}=\underset{w\in V^{*}}{\arg\max}\mathrm{Pr}\left(W|o\right)$$

Therefore, how to get the posterior probability, Pr(W|o), of the word sequence, W, given the observation vector sequence o, is the most critical problem.

The CTC uses a character sequence of length L, $C=\left\{c_{l}\in U|l=1,\cdots ,L\right\}$, to represent a possible word sequence. Here, U is a set of distinct characters. To deal with the repetition of character labels, the CTC defines an extra blank label, 〈b〉, to explicitly represent the character boundary. The enhanced character sequence $C^{\prime }$ with the label 〈b〉 is defined as:

(2) $$C^{\prime }=\left\{\langle b\rangle ,c_{1},\langle b\rangle ,c_{2},\cdots ,c_{L},\langle b\rangle \right\}=\left\{c_{l}^{\prime }\in U\cup \{\langle b\rangle \}|l=1,\cdots ,2L+1\right\}$$

The posterior probability, Pr(C|o), can be calculated by Equation (3):

(3) $$\mathrm{Pr}\left(C|o\right)=\sum_{z}\mathrm{Pr}\left(C|Z,o\right)\mathrm{Pr}\left(Z|o\right)\approx \underset{≜{\mathrm{Pr}}_{ctc}(C|o)}{\underbrace{\sum_{z}\mathrm{Pr}\left(C|Z\right)\mathrm{Pr}\left(Z|o\right)}}$$

where $Z=\left\{z_{t}\in U\cup \{\langle b\rangle \}|t=1,\cdots ,T\right\}$ is a character sequence with the label 〈b〉, which has the same length with the corresponding observation vector sequence o.

CTC obtains Equation (3) by using a conditional independence assumption, which can simplify the dependence between the character model, Pr(C|Z), and the acoustic model, Pr(Z|o), in CTC. ${\mathrm{Pr}}_{ctc}(C|o)$ is the objective function of CTC, and will be used in a later equation.

### 3.2. Attention-Based Model

Unlike CTC, the attention-based model estimates the posterior probability without the assumption of the condition independence, such as Equation (4).

(4) $$\mathrm{Pr}\left(C|o\right)=\underset{≜{\mathrm{Pr}}_{att}(C|o)}{\underbrace{\prod_{l=1}^{L}\mathrm{Pr}\left(c_{l}|c_{1},\cdots ,c_{l-1},o\right)}}$$

where ${\mathrm{Pr}}_{att}(C|o)$ is an objective function based on the attention mechanism. ${\mathrm{Pr}}_{att}\left(c_{l}|c_{1},\cdots ,c_{l-1},o\right)$ is calculated by Equations (5)–(8).

(5) $$h_{t}=\mathrm{Encoder}(o)$$

(6) $$a_{lt}=\left\{ \begin{array}{l} \mathrm{Contentattetion}\;\left(q_{l-1},h_{t}\right)\\ \mathrm{Locationattention}\;\left({\left\{a_{l-1}\right\}}_{t=1}^{T},q_{l-1},h_{t}\right) \end{array}\right.$$

(7) $$r_{l}=\sum_{t=1}^{T}a_{lt}h_{t}$$

(8) $$\mathrm{Pr}\left(c_{l}|c_{1},\cdots ,c_{l-1},o\right)=\mathrm{Decoder}\left(r_{l},q_{l-1},c_{l-1}\right)$$

where Equations (5) and (8) are encoder and decoder networks, respectively. Here $h_{t}$ is the output hidden vector of the encoder, and $c_{l}$ is the output character of the decoder. Attention weight $a_{lt}$ in Equation (6) is used to denote the soft alignment of $h_{t}$. The hidden vector $r_{l}$ in Equation (7) is the weighted sum of $h_{t}$. Contentattention(·) and Locationattention(·) in Equation (6) are content-based attention mechanisms with and without convolutional features, respectively [59]. The decoder network in (8) is a recursive network which takes the previous output $c_{l-1}$, hidden vector $q_{l-1}$, and hidden vector $r_{l}$, as conditions.

### 3.3. Hybrid CTC/Attention Architecture

A hybrid CTC/attention architecture is adopted in the end-to-end ASR of Mandarin. The advantages of a CTC and attention mechanism are fully utilized in the process of encoding and decoding.

**Figure 3:**
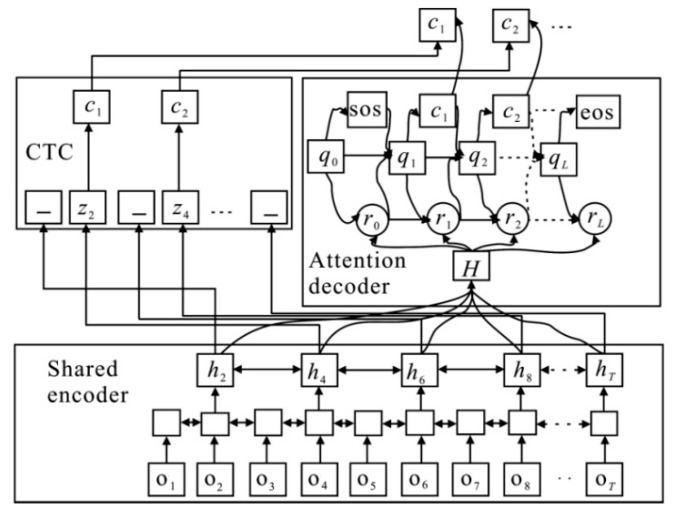
An overall framework of the end-to-end automatic speech recognition (ASR) system based on hybrid CTC/attention architecture.

The shared encoder uses CTC criterion and attention mechanisms for joint training, and the observation vector sequence, $\left\{o_{t}\cdots o_{T}\right\}$, is converted into the advanced feature sequence, $H=\{h_{t}\cdots h_{T}\}$. Then a character sequence, $\left\{c_{1}\cdots c_{l}\right\}$, is generated by the attention-based decoder. Label 〈sos〉 and 〈eos〉 are used to represent the beginning and end of the sequence, respectively. The overall framework of the end-to-end ASR based on hybrid CTC/attention architecture is illustrated in Figure 3.

In [14,15], a multi objective learning (MOL) framework is adopted. Among them, the attention-based method is the main method, and the CTC method is the auxiliary method for robustness. The CTC ensures the accurate alignment between the observation vector sequence and the character sequences during training. Within the MOL framework, the new objective, $L_{mol}$, is an interpolation of the CTC objective, $L_{ctc}$, and the attention objective, $L_{att}$. It should be noted that $L_{ctc}$ and $L_{att}$ are the logarithm of ${\mathrm{Pr}}_{ctc}(C|o)$ in Equation (3) and ${\mathrm{Pr}}_{att}(C|o)$ in Equation (4) respectively.

(9) $$L_{mol}=\alpha L_{ctc}+(1-\alpha )L_{att}$$

(10) $$L_{ctc}=\log{\mathrm{Pr}}_{ctc}(C|o)$$

(11) $$L_{att}=\log{\mathrm{Pr}}_{att}(C|o)$$

where α is a tunable parameter, which satisfies 0≤α≤1. When α=0, the objective to be maximized is the attention objective, and when α=1, it is the CTC objective.

However, in [14,15], the parameter α, used for linear interpolation, needs to be set manually before the beginning of training and remains unchanged throughout the training process. Despite its shortcomings, a dynamic parameter adjustment method is introduced in this paper.

(12) $$\alpha =\mathrm{sigmoid}(L_{ctc}-L_{att})$$

This parameter, α, which does not need to be set manually before training, can be adjusted continuously during training and helps estimate the alignment process better. When $L_{ctc}$ is greater than $L_{att}$, and α is greater than 0.5, the contribution of $L_{ctc}$ in Equation (9) is strengthened, and the contribution of $L_{att}$ is inhibited. When $L_{ctc}$ is less than $L_{att}$, and α is less than 0.5, the contribution of $L_{att}$ in Equation (9) is strengthened, and the contribution of $L_{ctc}$ is inhibited.

In the decoding process, a one-pass beam search algorithm is used to combine attention-based and CTC probability logarithm scores and perform joint decoding to further eliminate irregular alignment.

Assuming $c_{n}$ is the $n^{th}$ output given the history outputs, $c_{1:n-1}$, and the output of the encoder, $h_{1:T’}$, a linear combination of attention-based and CTC probability logarithm scores is performed during one-pass beam search.

(13) $$\log{\mathrm{Pr}}_{mol}\left(c_{n}|c_{1:n-1},h_{1:T{}^{\prime }}\right)=\alpha \log{\mathrm{Pr}}_{ctc}\left(c_{n}|c_{1:n-1},h_{1:T{}^{\prime }}\right)+(1-\alpha )\log{\mathrm{Pr}}_{att}\left(c_{n}|c_{1:n-1},h_{1:T{}^{\prime }}\right)$$

Here, $c_{1},\cdots ,c_{n}$ in ${\mathrm{Pr}}_{att}\left(\cdot \right)$, ${\mathrm{Pr}}_{ctc}\left(\cdot \right)$ and ${\mathrm{Pr}}_{mol}\left(\cdot \right)$ are different, corresponding to the $n^{th}$ output of the attention-based decoder, the CTC decoder, and the mixed decoder with the MOL framework respectively, as shown in Figure 3.

## 4. Experiments and Results

### 4.1. Databases

There are two kinds of experimental databases, the first being standard speech corpora, which are used to train standard acoustic models, and the other being learners’ non-standard speech corpora with experts’ detailed annotations, which are used to train and evaluate APED models.

#### 4.1.1. Standard Speech Corpora

CCTV: China Central Television (CCTV) news speech corpus. To train a standard acoustic model based on phones (in this paper, phones refer specifically to initials and finals in Mandarin, as detailed in Table 1), 186 audio segments of CCTV news broadcasting programs were collected, and speech data for nearly 70 h (16KHz sampling, 16bit quantization, sentence level segmentation, WAV format storage) were collected and corresponding texts (Chinese characters and Pinyin) were labeled manually. Among them, there are 17,359 sentences of male announcers and 15,931 sentences of female announcers. The male announcers are Luo Jing, Wang Ning, Zhang Hongmin, Kang Hui, and Guo Zhijian. The female announcers are Li Ruiying, Li Xiuping, Xing Tinbin, Hai Xia, and Li Zimeng. The number of sentences per announcer is shown in Table 2.

PSC-G1-112: A spot speech corpus (16KHz sampling, 16bit quantization, WAV format storage) of the 112 college students was collected in a PSC (Putonghua proficiency test), which is a state-level test in China. These students’ certification levels were both first class and second level, and there was confirmed to be no pronunciation errors and/or pronunciation defects after a careful manual check. Among them, the proportion of males to females (45 males and 67 females) is approximately balanced. Each student’s speech sample contains 100 monosyllabic words and 50 disyllabic words (including Erhua, also called retroflex suffixation), for a total of 204 syllables (Erhua is treated as a simple final er).

#### 4.1.2. Non-Standard Speech Corpora

PSC-1176: A spot speech corpus (16KHz sampling, 16bit quantization, WAV format storage) of the 1176 college students (567 males and 609 female) was collected in a PSC. The proportion of males to females was approximately balanced. Each student’s speech sample contains 100 monosyllabic words and 50 disyllabic words (including Erhua), for a total of 204 syllables (Erhua is treated as a simple final er).

Adhering to the scoring rules of the PSC, three national-level certified raters graded all phones in the corpus and marked all pronunciation defects and pronunciation errors in detail using our self-developed PSC scoring assistant software. Each initial, final, tone and Erhua of each syllable were respectively marked when they were found to be pronunciation errors or defects, and the real initial, final, tone and Erhua were also recorded in detail when they were distinguishable. The speech corpus met the requirement of training and testing of the pronunciation evaluation model, error detection model, and pronunciation diagnosis model. Three certified raters graded every phone, and we further integrated the three scores (determined by whether a phone is a pronunciation error) by voting.

For the experimental requirements, the PSC-1176 speech corpus was randomly divided into three parts, without duplication, and the proportion of males to females was approximately balanced. Each part consisted of 1000, 89, and 87 college students and they were marked as PSC-Train-1000, PSC-Test-89, and PSC-Develop-87, respectively. They were used as the training set, test set, and development set for the subsequent experiments. Their statistical information is shown in Table 3.

### 4.2. Experimental Configuration

We selected some of the most landmark conventional APED models as baseline systems of our proposed end-to-end APED model, which helped us analyze and compare their performance, advantages, and disadvantages. The GOP algorithm based on the GMM–HMM model in [4] was used as our first baseline system, which was named GMM_HMM_GOP. In [4], the concept of GOP and its robust calculation methods were proposed for the first time, and an APED was realized by pre-set thresholds. We used the algorithm based on the DNN–HMM model in [2] as our second baseline system, which was denoted as DNN_HMM_GOP. In [2], the GOP algorithm was redefined on the DNN–HMM framework for the first time, and the approximate GOP algorithm based on the senone was proposed to improve the robustness of the system. We used the AGPM, designed with a DNN–DNN framework, in [9] as our third baseline system, which was denoted as DNN_DNN_AGP. The AGPM could simultaneously integrate acoustic features of speech segments, corresponding graphemes, and canonical transcriptions through multi-distribution DNN, and could effectively model grapheme-to-likely pronunciation and phone-to-likely-pronunciation conversions in non-native speech. It achieved the best performance of all known algorithms on the non-native corpus used by the author. Our end-to-end system based on hybrid CTC/attention architecture was marked as CTC_Attention.

For the configurations of baseline systems, please refer to the respective literature. It should be noted that, due to the different speech corpora, different languages and different pronunciation units used in evaluation, the baseline systems are only adopted by the algorithms proposed in relevant literature, but the configurations are slightly different from those in literature. The configuration of our CTC_Attention is shown in Table 4.

### 4.3. Experimetal Performance Evaluation Metrics

#### 4.3.1. Performance of ASR Systems

The word error rate (WER) is the most important metric to evaluate the performance of ASR systems. We were mainly concerned about recognition and detection performance at the phone level in our work. Therefore, we used the phone error rate (PER) as the performance evaluation metrics of ASR systems. Like the WER, the PER is calculated by Equation (14).

(14) $$\mathrm{PER}=\frac{S+D+I}{N}$$

where *N* is the total number of phones. *S*, *D*, and *I* denote the counts of substitution errors, deletion errors, and insertion errors, respectively, and they were obtained through Needleman–Wunsch algorithm [58] to compare speech recognition results with canonical transcriptions.

#### 4.3.2. Performance of APED systems

**Figure 4:**
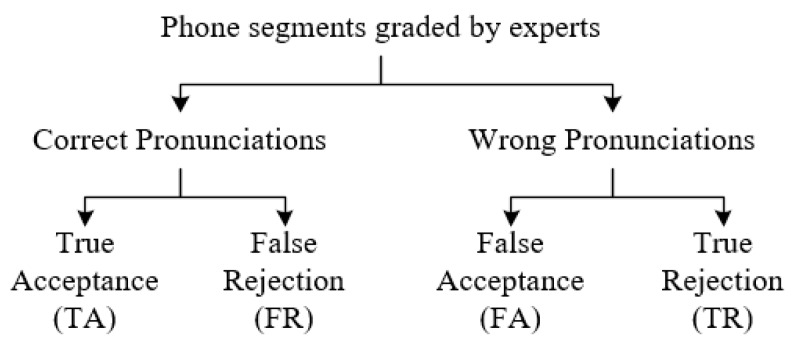
The hierarchical evaluation structures.

APED can be achieved by comparing the recognized phone sequences with the canonical transcriptions, and the PER is also one of the most important metrics to evaluate in the performance of the APED. For more detailed experimental results and performance comparisons, the hierarchical evaluation structure illustrated in Figure 4 is proposed in [60], which has also been used in [61].

In Figure 4, phone segments are marked (graded) as correct pronunciations and wrong pronunciations by experts according to their pronunciations. True acceptance (TA) means the phone segments were marked by experts and recognized by the ASR system as the correct pronunciation, true rejection (TR) refers to phone segments marked as wrong pronunciations by experts and identified as incorrect by the ASR system. False rejection (FR) refers to phone segments recognized as wrong pronunciations when the actual pronunciations are correct, false acceptance (FA) refers to phone segments misclassified as correct but were actually mispronounced.

Therefore, TA and FR are correct pronunciations, while FA and TR are wrong pronunciations. For the APED task, TA and TR are the correct outcomes, whereas FR and FA are the incorrect outcomes. FR is more harmful than FA, and TR is more meaningful than TA in the practical CAPT system.

We can first get the alignment results of ASR (i.e., C, S, D, and I) by comparison to the canonical phone sequence in the reference transcription with the phone sequence recognized by ASR. After this, we identify the type (i.e., TA, FA, FR, and TR) of each phone in the APED task according to the alignment results of ASR (i.e., C, S, D, and I) and the results marked by experts. The process is shown in Table 5. Finally, we can count the number of TA, FA, FR, and TR, and further calculate other metrics to evaluate the performance of APED.

TA means that the phone segment, which is marked T by experts, is recognized correctly by the ASR (the result analysis is marked C). FA means that the phone segment, which is marked F by experts, is recognized correctly by the ASR (the result analysis is marked C). FR means that the phone segment, which is marked T by experts, is not recognized correctly by the ASR (the result analysis is marked S, D, or I). TR means that the phone segment, which is marked F by experts, is not recognized correctly by the ASR (the result analysis is marked S, D, or I).

The false rejection rate (FRR) and false acceptance rate (FAR) are widely used as the performance measures for APED tasks [1,62]. They are calculated through Equations (15) and (16), respectively.

(15) $$\mathrm{FRR}=\frac{\mathrm{FR}}{\mathrm{TA}+\mathrm{FR}}$$

(16) $$\mathrm{FAR}=\frac{\mathrm{FA}}{\mathrm{FA}+\mathrm{TR}}$$

where TA, FR, FA, and TR are the total number of phone segments for each group in Figure 4.

Besides FRR and FAR, precision, recall, and F-measure are also standard metrics to evaluate the performance of the APED system [60,61]. They are defined as follows:

(17) $$\mathrm{Precision}=\frac{\mathrm{TR}}{\mathrm{TR}+\mathrm{FR}}$$

(18) $$\mathrm{Recall}=\frac{\mathrm{TR}}{\mathrm{TR}+\mathrm{FA}}=1-\mathrm{FAR}$$

(19) $$\mathrm{F-measure}=2\frac{\;\mathrm{Precision}\;*\;\mathrm{Recall}\;}{\;\mathrm{Precision}\;+\;\mathrm{Recall}\;}$$

In addition, the accuracies of APED systems are calculated by Equation (20):

(20) $$Accuracy=\frac{\mathrm{TA}+\mathrm{TR}}{\mathrm{TA}+\mathrm{FR}+\mathrm{FA}+\mathrm{TR}}$$

### 4.4. Experimental Results and Discussion

#### 4.4.1. ASR Tasks

**Figure 5:**
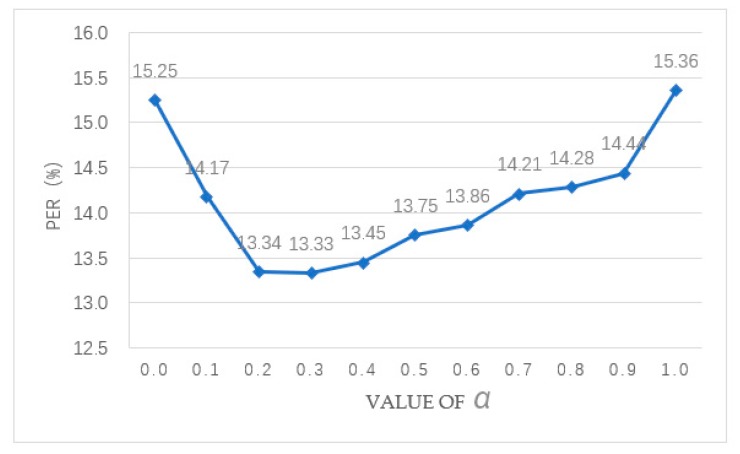
Effect of the hyper-parameter α in the hybrid model.

Firstly, we compare the performance of ASR based on the CTC model, the attention-based model and the CTC/Attention hybrid model using the Mandarin ASR task. A two-layer BLSTMP is chosen as the encoder, and the number of cells in each layer is 256. The attention mechanism includes content-based attention and location-aware attention, and [15] can be referred to for further details. The detailed experimental results are shown in Table 6. Because of the complementarity of the CTC model and the attention-based model, the hybrid model can effectively increase the alignment effect of the attention-based model and reduce the PER when the hyper-parameter α takes a different value. In essence, α is used to represent the proportion of the CTC model and the attention-based model in the hybrid model, and it has a significant influence on the performance of the hybrid model. When α is set to 0.0, the hybrid model degenerates to the CTC model, while when α is set to 1.0, the hybrid model degenerates to the attention-based model. When α is set to 0.2, 0.3, and 0.4, the PER of the hybrid model is relatively low at 13.34, 13.33, and 13.45, respectively. The lowest PER is 13.33 when α is set to 0.3. The best hybrid model (α=0.3) can reduce the PER from 15.36 to 13.33, a relative reduction of 13.22%, compared to the CTC model (α=1.0). And it can reduce the PER from 15.25 to 13.33, a relative reduction of 12.59%, compared to the attention-based model (α=0.0). So, the hybrid model is obviously more effective than the CTC model and the attention-base model. The influence of the hyper-parameter α on the performance for the hybrid model can be seen more clearly in Figure 5. However, the disadvantage of α is that it must be set manually before the beginning of training and remain unchanged throughout the training process. Therefore, we propose a new dynamic adjustment method to *α* in Section 3.3. The hybrid model can reduce the PER from 13.33 to 13.01, a relative reduction of 2.40%, when α is set from the optimal value 0.3 to the dynamic adjustment value obtained from Equation (12).

The key to improve the modeling ability of BLSTMP is to increase the number of layers. For the three different ASR systems above, we set the number of layers in their BLSTMP encoders to two, three, four, and five respectively, and their performance is shown in Table 7. For the ASR system based on the improved CTC/attention hybrid architecture (α dynamic adjustment), when the number of layers increases from two to four, the PER decreases from 13.01 to 10.25, a relative decrease of 21.21%. When the number of layers increases to five, the PER begins to rise. The same is true for the ASR system based on the CTC model, and the ASR system based on attention model. This is mainly due to the lack of data for the training of network parameters, which leads to under-fitting results. Therefore, in subsequent experiments, CTC_Attention refers to the ASR system based on the improved CTC/attention hybrid architecture with a four-layer BLSTM encoder and dynamic parameter adjustment.

In the same case, we continue to compare the performance of ASR systems with different model architectures. As DNN is a discriminant model, the accuracy of the model will generally be higher. The ASR system based on DNN–HMM is significantly higher than the one based on GMM–HMM in the performance, as the PER almost drops by half, from 28.64 to 12.79. Although our CTC/attention hybrid model performs slightly worse than the DNN–DNN model, the CTC/Attention hybrid model does not require the accurate segmentation of phone boundaries and does not need to train multiple models in turn, so the system based on the CTC/attention hybrid model is more simple and convenient to build. The experimental results of ASR systems with different model architectures are shown in Table 8.

#### 4.4.2. APED Tasks

Next, we compare the performance of different models for APED tasks in the test set. From Table 9, we can see that GMM_HMM_GOP uses the monophonic acoustic model and the standard GOP algorithm. The performance is still relatively low, and its accuracy is only 70.55. DNN_DNN_AGP considers the acoustic features, as well as adjacent phone and character labels, and uses the DNN discriminant model to achieve the highest accuracy, reaching 90.38. Our CTC_Attention also obtains the second highest accuracy, reaching 90.14. The gap between CTC_Attention and DNN_DNN_AGP is very small in regards to the accuracy, showing a 0.2% difference only. Moreover, the F-measure of CTC_Attention is 67.39, the highest of all systems. This shows that CTC_Attention has a high precision and recall rate for pronunciation errors and is more suitable for the APED task.

To compare the characteristics of different models, we focus on the performance of these systems based on different model architectures on different phones (initials and finals). The total number and proportion of pronunciation errors of different phones are usually different in the corpus. For convenience of comparison, we present the performance of each system for four phones: zh, g, ang, and a, respectively. The results can be seen in Table 10, Table 11, Table 12 and Table 13. For phones zh, g, ang, and a, the error rates of their pronunciation in PSC-Test-89 are 32.12%, 9.37%, 28.95%, and 10.47%, respectively.

The higher the error rate of phone pronunciation, the more difficult or error-prone the phone is. The greater the level of confusion with other phones, the less recognizable it is, and the more refined model is needed.

**Figure 6:**
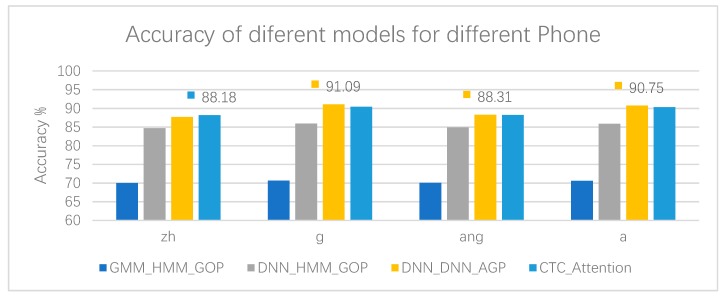
Accuracy of different models for four phones, zh, g, ang, and a.

**Figure 7:**
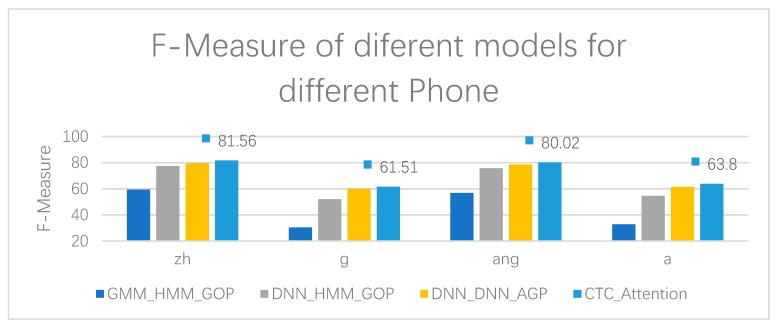
F-Measure of different models for four phones, zh, g, ang, and a.

As can be seen from Table 10, Table 11, Table 12 and Table 13, DNN_DNN_AGP has the highest accuracy in terms of initial g, final ang, and final a, regardless of the pronunciation error rate of the phone. Our CTC_Attention also has very high accuracy, and achieves the highest value in the initial zh, and the highest F-Measure in the initial z, initial g, final ang, and final a, indicating that CTC_Attention has a better comprehensive performance in the precision and recall of pronunciation errors. The results of the experiment, the accuracy, and F-Measure of different models for four phones, are shown more clearly in Figure 6 and Figure 7, respectively.

#### 4.4.3. Discussion of Pitch Features

Mandarin is a tonal language and adding a pitch feature is usually beneficial to improve recognition results in the ASR task. Because of this, pitch features were added as part of the input features in CTC_Attention. To detect whether the pitch features improved the performance of the system, we removed the pitch features in CTC_Attention. We found that the performance of neither the ASR system nor the APED system had obvious change without pitch features. In the ASR task, PER decreased slightly, by 0.09% after adding pitch features, as shown in Table 14. In the APED task, the accuracy increased slightly by about 0.02% after adding pitch features, but the F-Measure decreased from 67.50 to 67.39, as shown in Table 15. Therefore, it is not necessary to add pitch features in phone recognition and phone pronunciation error detection.

## 5. Conclusions and Prospect

From the perspective of the development of ASR technology, this paper carefully considers the classical methods, technical routes, and technical iteration process of APED technology over the past 20 years to help us analyze and compare the performance, advantages, and disadvantages, as well as the inheritance and applicability of different models. Furthermore, we proposed a new end-to-end ASR system based on improved hybrid CTC/attention architecture. The complementarity of CTC and attention is fully utilized to improve the performance of the ASR system, and then it is directly applied to an end-to-end APED task. It is no longer necessary to force alignment and segmentation of audio speech, nor does it require multiple complex models, such as a language model and a pronunciation dictionary. Our model is a suitable general solution for L1-independent CAPT. Moreover, we find that on the accuracy metrics, our ASR system based on the improved hybrid CTC/attention architecture (CTC_Attention) is close to the state-of-the-art ASR system based on the DNN–DNN architecture (DNN_DNN_APG) and has a stronger effect on the F-measure metrics, which are especially suitable for the requirements of the APED task.

In addition, with the development of technology, there is still a lot of work worth studying.

1. We found that pitch features have little effect on our improved CTC/attention hybrid model for the phone-level ASR and APED tasks. However, we all know that effective features play an important role in these tasks. Deep learning is a type of representation learning technology, suitable for feature extraction in particular. It is a feasible idea, then, to extract more effective features directly from the speech spectrum using deep learning models (such as CNN).
2. Transformer is a new network based on the self-attention mechanism and has achieved great success in neural machine translation (NMT) and other natural language process (NLP) tasks. Since the outstanding performance of Transformer was observed, it has been extended to speech as its basic architecture, and the Transfomer-based ASR has also achieved excellent results [63,64]. It shows excellent performance in embedding the position information in speech features, encoding relationships between local concepts within a long range, and effectively recovering these relationships during decoding. Therefore, it is worth looking at using Transfomer to build an APED system in the future.
3. Multi-task learning (MTL) [65] improves learning efficiency and model generalization for the task-specific models. Several related tasks learn at the same time, and all of these tasks usually share a part of the representation. Each new task contributes to the model learning by adding information and transferring knowledge. The MTL approach is applied to neural networks by sharing some of the hidden layers between different tasks. Some research could improve the accuracy of CTC-based ASR by incorporating acoustic landmarks, which could help CTC training converge more rapidly and smoothly [66,67]. Moreover, the information of acoustic landmarks could be obtained, which could be used as an additional information source, to further improve the performance of the APED system [68]. Similarly, through the MTL’s articulatory features, the APED system not only improves in accuracy, but also obtains the auxiliary articulatory information which may help us to provide specific and easy operative feedback. Examples of this could include tips, such as “open your mouth wider”, or “put your tongue in a lower position”.

## Figures and Tables

**Table 1:** A list of initials and finals in Mandarin.

Type	Quantity	Phone Units
Initial	21	b p m f d t n l g k h j q x zh ch sh r z c s
Simple final	9	a o e i u ü -i1 -i2 er
Compound final	13	ai ei ao ou ia ie ua uo üe iao iu uai ui
Final with a nasal ending	16	an ian uan üan en in un ün ang iang uang eng ing ueng ong iong

Note: There are 39 finals in Chinese Pinyin defined by linguistic phoneticists. The symbols -i1 and -i2 are respective of the simple final which can follow only the initials zh, ch, sh, and z, c, s, but not any other initials. Although ê is also a simple final in Chinese Pinyin, it is not independently syllabled. It is always combined with i and ü to form the compound final ie and üe, so it is not placed in the simple final list. In conclusion, there are 59 total phones, including 21 initials, and 38 finals in this paper.

**Table 2:** Number of sentences of announcers in China Central Television (CCTV) news speech corpus.

**Male** **Announcer**	**Luo Jing**	**Wang Ning**	**Zhang Hongmin**	**Kang Hui**	**Guo Zhijian**	**Total**
**Number of sentences**	5131	5468	4195	1884	681	17,359
**Female Announcer**	**Li Ruiying**	**Li Xiuping**	**Xing Tinbin**	**Hai Xia**	**Li Zimeng**	**Total**
**Number of sentences**	5268	5349	4657	425	232	15,931

**Table 3:** Phone tokens for correct and incorrect pronunciations on different datasets.

Data Collection	Phones in Total	Phones with Pronunciation Error	Pronunciation Error Rate %
Training Set PSC-Train-1000	408,000	50,616	12.41%
Develop Set PSC-Develop-87	35,496	4432	12.49%
Test Set PSC-Test-89	36,312	4544	12.51%
Total	479,808	59,592	12.42%

**Table 4:** Experimental configuration of CTC Attention.

Acoustic Unit	Mono-Phone (Initial and Final in Mandarin)
**Acoustic Feature**	The window length is 30 ms and the frame shift is 30 ms. The input feature is a 40-dimensional filter bank with first-order and second-order derivatives, as well as a 3-dimensional pitch.
**Configuration**	The output of the CTC is 59 units, including 58 labels of initials and finals and one blank label. Because CTC does not need a context decision tree to achieve good performance, mono phone (initial or final) is taken as the acoustic unit. The lower frame rate can reduce the computational cost of the decoding process and greatly improve the decoding speed. The input of the attention-based model is the same as the CTC, and the encoder is shared. The output of attention-based model is 60 units, including 58 phone labels and < SOS > < EOS >. In the decoding process, the irregular alignment can be further eliminated by combining the probability score based on the attention and CTC in the one-pass beam search algorithm. CCTV, PSC-G1-112, and PSC-Train-1000 speech corpora are used as training data sets. Finally, the performance is tested in the PSC-Test-89 speech corpus.

**Table 5:** A result analysis of APED Based on ASR.

Canonical Phone in the Reference Transcription	*p*	*p*	*p*	*p*	*p*	*p*		
**Phone Recognized by ASR**	*p*	*p*	*q*	*q*			*p*	*p*
**Result analysis of ASR**	C	C	S	S	D	D	I	I
**Marked by expert**	T	F	T	F	T	F		F
**Result analysis of APED**	TA	FA	FR	TR	FR	TR	FR	TR

Note: the phones, marked *p* and *q* in this table, refer to two different phones in the phone set. The result analysis of ASR includes four cases: C (correct), S (substitution error), D (deletion error) and I (insertion error). The result marked by experts includes two cases: T (correct pronunciation) and F (pronunciation error). Result analysis of APED includes four cases: TA (true acceptance) and FR (false rejection), FA (false acceptance) and TR (true rejection).

**Table 6:** Performance of ASR systems with different end-to-end models.

Name	PER %
CTC	15.36
Attention	15.25
CTC_Attention (α=0.1)	14.17
CTC_Attention (α=0.2)	13.34
CTC_Attention (α=0.3)	13.33
CTC_Attention (α=0.4)	13.45
CTC_Attention (α=0.5)	13.75
CTC_Attention (α=0.6)	13.86
CTC_Attention (α=0.7)	14.21
CTC_Attention (α=0.8)	14.28
CTC_Attention (α=0.9)	14.44
CTC_Attention (α dynamic adjustment)	**13.01**

Note: the phone segments marked wrong pronunciations by experts in the test set are ignored when the phone error rate (PER) of ASR is calculated.

**Table 7:** Performance of ASR systems when the number of layers in their bidirectional long short-term memory projection (BLSTMP) encoders is different.

Name Number of Layers	PER %			
	2	3	4	5
CTC	15.36	14.25	13.34	14.28
Attention	15.25	13.79	13.06	13.87
CTC_Attention (α dynamic adjustment)	13.01	11.24	**10.25**	11.43

**Table 8:** Performance of ASR systems with different model architectures.

Name	PER %
GMM_HMM_GOP	28.64
DNN_HMM_GOP	12.79
DNN_DNN_AGP	**10.17**
CTC_Attention	10.25

**Table 9:** Performance evaluation of different APED systems for all initials and finals in Mandarin.

	FRR	FAR	Precision	Recall	F-Measure	Accuracy
GMM_HMM_GOP	29.10	31.89	25.07	68.11	36.65	70.55
DNN_HMM_GOP	13.57	18.86	46.09	81.14	58.79	85.77
DNN_DNN_AGP	5.85	35.97	61.01	64.03	62.48	**90.38**
CTC_Attention	8.62	18.55	57.47	81.45	**67.39**	90.14

**Table 10:** Performance of APED systems for initial zh.

	FRR	FAR	Precision	Recall	F-Measure	Accuracy
GMM_HMM_GOP	29.10	31.91	52.55	68.09	59.32	70.00
DNN_HMM_GOP	13.57	18.90	73.88	81.10	77.32	84.72
DNN_DNN_AGP	5.85	26.00	85.69	74.00	79.42	87.68
CTC_Attention	8.62	18.59	81.72	81.41	**81.56**	**88.18**

**Table 11:** Performance of APED systems for initial g.

	FRR	FAR	Precision	Recall	F-Measure	Accuracy
GMM_HMM_GOP	29.10	31.91	19.48	68.09	30.29	70.64
DNN_HMM_GOP	13.57	18.89	38.19	81.11	51.93	85.93
DNN_DNN_AGP	6.85	28.82	51.79	71.18	59.96	**91.09**
CTC_Attention	8.62	18.57	49.42	81.43	**61.51**	90.45

**Table 12:** Performance of APED systems for final ang.

	FRR	FAR	Precision	Recall	F-Measure	Accuracy
GMM_HMM_GOP	29.11	31.92	48.8	68.08	56.85	70.08
DNN_HMM_GOP	13.57	18.89	70.89	81.11	75.66	84.89
DNN_DNN_AGP	5.86	26.01	83.74	73.99	78.56	**88.31**
CTC_Attention	8.99	18.58	78.67	81.42	**80.02**	88.23

**Table 13:** Performance of APED systems for final a.

	FRR	FAR	Precision	Recall	F-Measure	Accuracy
GMM_HMM_GOP	29.10	31.90	21.49	68.10	32.67	70.61
DNN_HMM_GOP	13.57	18.91	41.13	81.09	54.58	85.87
DNN_DNN_AGP	6.85	29.80	54.53	70.20	61.38	**90.75**
CTC_Attention	8.62	18.62	52.46	81.38	**63.80**	90.33

**Table 14:** Performance Comparison of ASR systems before and after adding pitch features.

Input Features	PER
Filterbank	10.26
Filterbank + pitch	10.25

**Table 15:** Performance Comparison of APED systems before and after adding pitch features.

Input Features	FRR	FAR	Precision	Recall	F-Measure	Accuracy
Filterbank	8.72	17.99	57.35	82.01	**67.50**	90.12
Filterbank + pitch	8.62	18.55	57.47	81.45	67.39	**90.14**
